# Revolutionizing Cardiovascular Health with Nano Encapsulated Omega-3 Fatty Acids: A Nano-Solution Approach

**DOI:** 10.3390/md22060256

**Published:** 2024-05-30

**Authors:** Richa Gill, Mashael Al-Badr, Mohammad Alghouti, Nura Adam Mohamed, Haissam Abou-Saleh, Md Mizanur Rahman

**Affiliations:** 1 Biological Science Program, Department of Biological and Environmental Sciences, College of Arts and Sciences, Qatar University, Doha P.O. Box 2713, Qatar; rg1703022@student.qu.edu.qa (R.G.); malbadr@qu.edu.qa (M.A.-B.); 2 Environmental Science Program, Department of Biological and Environmental Sciences, College of Arts and Sciences, Qatar University, Doha P.O. Box 2713, Qatar; mohammad.alghouti@qu.edu.qa; 3 Biomedical Research Center, Qatar University, Doha P.O. Box 2713, Qatar; nura.adam@qu.edu.qa; 4 Biomedical Sciences Department, College of Health Sciences, Qatar University, Doha P.O. Box 2713, Qatar

**Keywords:** omega-3 fatty acids, cardiovascular disease, nanoparticle encapsulation, metal–organic framework, drug delivery systems

## Abstract

Omega-3 polyunsaturated fatty acids (ω-3 PUFAs) offer diverse health benefits, such as supporting cardiovascular health, improving cognitive function, promoting joint and musculoskeletal health, and contributing to healthy aging. Despite their advantages, challenges like oxidation susceptibility, low bioavailability, and potential adverse effects at high doses persist. Nanoparticle encapsulation emerges as a promising avenue to address these limitations while preserving stability, enhanced bioavailability, and controlled release. This comprehensive review explores the therapeutic roles of omega-3 fatty acids, critically appraising their shortcomings and delving into modern encapsulation strategies. Furthermore, it explores the potential advantages of metal–organic framework nanoparticles (MOF NPs) compared to other commonly utilized nanoparticles in improving the therapeutic effectiveness of omega-3 fatty acids within drug delivery systems (DDSs). Additionally, it outlines future research directions to fully exploit the therapeutic benefits of these encapsulated omega-3 formulations for cardiovascular disease treatment.

## 1. Introduction

Cardiovascular diseases (CVDs) remain the world’s leading cause of mortality, claiming a staggering 695,000 lives in the United States alone in 2021 [1], translating to roughly one death every 30 s [2]. According to a 2019 report by the World Health Organization, CVD contributes to 32% of total worldwide fatalities, with 85% of these fatalities attributed to heart attacks or strokes [3]. Major contributors and risk factors for CVD include elevated blood pressure, diabetes, and cholesterol levels, as well as smoking, unhealthy diet, obesity, and physical inactivity [4].

According to a recent report from the American Heart Association, an intake of approximately 3 g of omega-3 fatty acids daily, whether obtained from food or supplements, appears to be the optimal amount for reducing high blood pressure and preventing cardiovascular disease, as indicated by a review of multiple research studies [5]. Omega-3 fatty acids (ω-3 FAs) are categorized as polyunsaturated fatty acids (PUFAs) that include a minimum of a single double bond (C=C) between the carbon atoms in the third and fourth positions from the methyl end of the fatty acid [6,7]. PUFAs are long-chain fatty acids (LC-FAs) found in oily fish like sardines, tuna, and salmon, and other seafood like shellfish, algae, and shrimp, as well as particular plants and nut-based oils [8]. The most common bioactive ω-3 FAs are eicosapentaenoic acid (EPA) (C20:5 ω-3), docosahexaenoic acid (DHA) (C22:6 ω-3), and α-linolenic acid (ALA) (C18:3 ω) [9]. ALA can undergo various elongation and desaturation processes within the body to be converted into EPA and DHA [10]. However, the conversion rates of ALA to EPA and DHA may be relatively low, potentially insufficient to confer health benefits. Therefore, it is essential to ensure adequate intake of EPA and DHA for various aspects of health, including cardiovascular health, brain function, and inflammation regulation. PUFAs are essential nutrients that must be obtained from the diet because the body cannot synthesize them.

**Figure 1:**
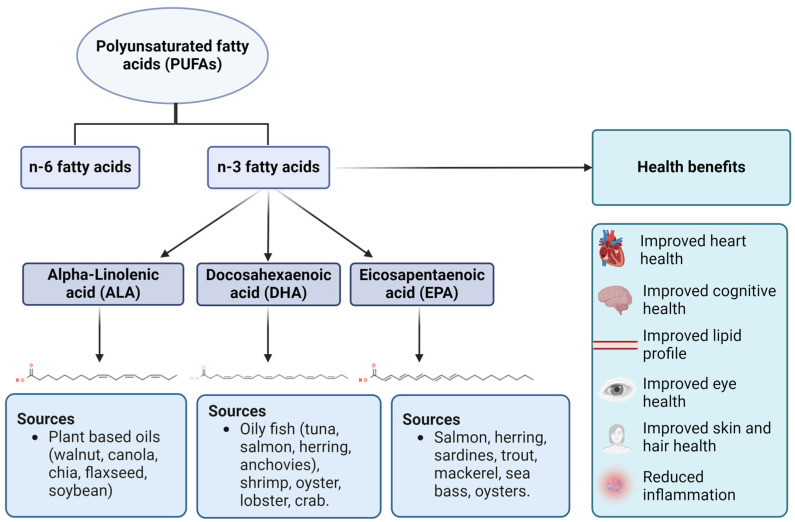
Types, sources, and health benefits of omega-3 fatty acids. Three types of n-3 fatty acids are ALA (plant-based sources), DHA (animal-based sources), and EPA (animal-based sources). As mentioned earlier, n-3 fatty acids have a wide range of health benefits related to cardiovascular, ocular, cognitive, and dermal health.

PUFAs play numerous physiological roles involving cell signaling and transmission, intercellular contact, membrane fluidity, phospholipid membrane maintenance, reduced inflammation [11,12], and improved fatty acid oxidation [13]. Hence, a deficiency in ω-3 FAs can deteriorate bone health [14,15,16], cardiovascular health [17], and skin and neurological health [13,18] (Figure 1). Along with this, ω-3 FA ingestion is associated with a lowered prevalence of inflammation [19] through the lowering of pro-inflammatory cytokines triggered by oxidative stress in macrophages isolated from female mice [20] and rats [21]. Various studies have also assessed the anti-inhibitory effect of ω-3 FAs on cancers in female mice [22] and cancer cell lines [23]. Over the decades, various preclinical and clinical studies have been conducted using ω-3 FAs to show their efficacy in reducing cardiovascular ailments. This review paper will provide an overview of ω-3 FAs with a focus on their effect on the cardiovascular system, along with shortcomings in the delivery of ω-3 FAs to which nanoparticles could be potentially a viable solution.

### 1.1. Preclinical Studies

Over the years, various pre-clinical studies conducted on a range of animal experimental units have proved the effectiveness of ω-3 FAs in cardioprotection and decreasing markers of cardiovascular stress, as summarized in Table 1. 

### 1.2. Clinical Studies

Several significant meta-analyses of ω-3 FAs and their connection to cardiovascular morbidity and mortality have been published in the last four decades. Some of these studies concluded that fish oil supplementation lowers the risk of cardiovascular events and the mortality rates from CVD, while others did not support this conclusion, as seen in Table 2.

The differences in research findings might be attributed to the various fatty acids employed in these studies, which ranged fromo-3 carboxylic acid formulation to ethyl esters of EPA, DHA, and ALA. The omega-3 CA employed in the STRENGTH study was EPA as a free fatty carboxylic acid, which has greater absorption in a diet low in fat than EPA and DHA ethyl esters, comparable to the EPA ethyl ester used in the REDUCE-IT study. Consequently, omega-3 CA increases blood levels of EPA and DHA when combined with a diet low in fat, but not with a standard diet. In the STRENGTH study, omega-3 CA was administered independent of eating habits or dietary fat composition, which might culminate in fluctuation in EPA and DHA levels in the blood and perhaps reduce the impact on cardiovascular events. Icosapent ethyl additionally contains approximately 25% higher EPA in each dose than omega-3 CA, resulting in 61% greater EPA levels in blood in REDUCE-IT versus STRENGTH from comparable baseline values [41].

### 1.3. Epidemiological Studies

Apart from clinical studies, epidemiological studies were also conducted on the effect of ω-3 FAs on the reduction of cardiovascular events. The results of the studies in Table 3 were mixed, with some claiming that ω-3 FAs decrease cardiovascular events and others claiming no such outcome.

### 1.4. Case-Control Studies

The number of case-control studies measuring EPA and DHA levels in plasma as a biomarker of ω-3 FA after 2004 declined due to the introduction of the new concept of the omega-3 index (O3I) [50]. The omega-3 index is defined as the proportion of EPA and DHA in a total of 26 distinct fatty acids in the membrane of red blood cells (RBCs), with many studies claiming that an O3I of 8% is required to elicit the cardioprotective effect of ω-3 FAs [51]. There are many reasons why measuring EPA and DHA in erythrocyte membranes is a more accurate method of assessing ω-3 FA intake in the diet. RBC fatty acid content has relatively little biological variability in comparison to plasma. Erythrocyte lipids are nearly entirely composed of phospholipids, and RBC fatty acid content represents tissue fatty acid composition [52]. Table 4 shows the results of a few of the earliest case-control studies.

## 2. Nanoparticles and Administration of Omega-3

### 2.1. Obstacles in the Effective Administration of Omega-3 PUFAs 

**Figure 2:**
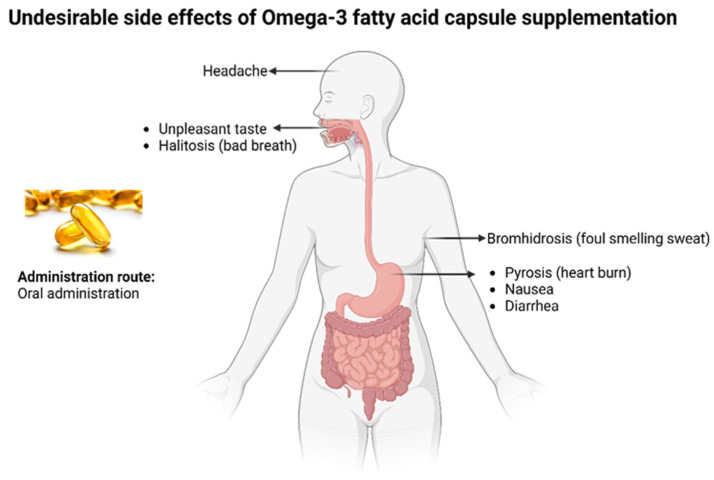
Common undesirable side effects of omega-3 fish oil capsule supplements.

There have been concerns regarding the optimal source and route of administration of ω-3 FAs for human consumption. Even though the simplest method of ω-3 FA intake is through eating fish, decreasing fish stocks worldwide and biomagnification of toxic trace elements due to water pollution are proving to be valid concerns [58]. An alternate method is consuming ω-3 FA supplements. The issue with supplements is that they may cause undesired side effects, such as stomach upsets, stale breath, nausea, etc. (Figure 2). Moreover, the release of ω-3 FAs in supplements occurs rapidly, simultaneously delivering the entire amount [59].

The bioavailability of long-chain ω-3 PUFA is another complex issue that needs to be tackled. Bioavailability is a relative term that refers to both the rate of absorption and the amount of the substance ingested [60]. Bioavailability encompasses both absorption speed and quantity absorbed. It is influenced by gastrointestinal absorption and transport rates to the portal system. In a broader sense, it considers the amount reaching systemic circulation or the intended destination. Understanding this aids in pharmacokinetics and dietary planning. NPs can significantly improve the pharmacokinetics of drugs by increasing their solubility [61], stability [62], permeability [63], half-life, and residence time [64]. The major cause of the limited bioavailability of long-chain n-3 PUFAs is low solubility in the aqueous gastrointestinal fluids of the GI tract, alongside their vulnerability to chemical breakdown during transit through the stomach [65]. Long-chain omega-3 PUFAs are mostly present in fish as triacylglycerides (TAGs), phospholipids (PLs), and free fatty acids (FFAs) [66]. ω-3 PUFAs, when ingested as pure oil, cannot be completely absorbed by the cells in our intestines, resulting in reduced bioavailability [67]. Fish oils are transformed into oil-in-water emulsions within the mouth and stomach, while emulsified oils are colloidal before consumption. Lipases in the stomach and pancreas subsequently attach to the surface of the lipid drop and begin the process of lipid digestion, which converts TAGs into FFAs and monoacylglycerols (MAGs). The digestive products, FFAs and MAGs, subsequently combine with bile and PLs to produce blended micelles that transport the lipids across the mucus membrane to the epithelial cell surfaces, where they are absorbed. Investigations have demonstrated that the bioavailability of ω-3 PUFAs is enhanced when consummated in an emulsified way rather than in a pure form [65]. Widely ingested edible oils and products have less ω-3 PUFAs compared to the suggested daily guideline; also, the minimal quantity of PUFAs taken is poorly absorbed. The transformation rate of plant-sourced ω-3 FAs, namely, ALA into EPA and DHA, the primary ω-3 PUFAs accountable for the reported benefits, is only 3–6%, with DHA having an inadequate transformation rate maxing out at 1% [68].

Apart from these complications, lipid oxidation is an additional problem that needs to be addressed. Among the different types of ω-3 FAs, EPA and DHA are extremely vulnerable to the oxidation of lipids due to the presence of numerous double bonds [69]. The oxidative degradation of lipids in fish oil, along with other PUFA-rich and fortified foods is a severe issue that frequently results in a decrease in shelf-life, customer acceptance, performance, nutrient content, and quality [70]. The oxidation of these ω-3 FAs results in aldehydes that are toxic to proteins and nucleic acids in the human body, namely 4-hydroxy-2-nonenal and malondialdehyde [71]. The toxicity stems from their capacity to crosslink proteins and attach covalently to nucleic acids. 

Lipid oxidation in fish oils is heightened by exposure to light, oxygen, and heat. Lipid oxidation creates three major issues: (i) it produces disagreeable unpleasant flavors, (ii) it decreases the nutritional content of lipid-containing food items, and (iii) free radicals generated during oxidation may contribute to the occurrence of atherosclerosis in the system [72]. Nevertheless, the low oxidative resistance of ω-3 PUFAs makes these oil-enriched foods require potent antioxidant defense to avert oxidative degradation and undesirable flavor formation [73]. Some solutions to stabilize ω-3 PUFAs include providing antioxidants for oxidative stability, mixing and blending with other oils, hydrogenation, and interesterification [68]. However, another innovative solution might be ω-3 FA encapsulation using nanoparticles as part of nanotechnology.

### 2.2. Nanoencapsulation of Omega-3 PUFAs

Nanoparticles (NPs) are the focal point of nanomedicine, which is a branch of medicine that relies on smart drug delivery technology that can boost the biological action, pharmacological index, and physiological half-life of the drug loaded inside the body [74]. Nanoparticles are increasingly becoming popular as drug delivery systems, especially to treat CVDs [75] and metabolic disorders [76] due to their ability to have a comparatively big surface area that can attach to, adsorb, and transport molecules, including drugs, probes, and protein molecules [77]. Furthermore, for the administration of medication, engineered nanoparticles, as well as the drug itself, can be synthesized at nanoscale and operate as a carrier for themselves [78]. There exists an array of nanoparticles, each having distinct features, advantages, disadvantages, and applications (Table 5).

In the past few years, multiple studies have been performed that utilized encapsulated ω-3 FAs within NPs as a drug delivery method. An in vitro study by Deshpande et al. tested a nanotechnology-focused method for administering ω-3 FAs to the walls of vascular vessels alongside other drugs to avoid occlusive vasculopathy after vascular injury [107]. The researchers created an ω-3-FA-rich oil-in-water nanoemulsion composition using flax seed oil naturally rich in ALA, that was administered to cultured vascular cell lines. Combining the administration of 17-βE and CER-laden nanoemulsions had a stronger anti-proliferative impact on vascular smooth muscle cells as compared to endothelial cells [108]. In 2016, the same researchers set out to examine the effectiveness of an ω-3 FA containing 17-βE nanodelivery setup in treating induced atherosclerosis. The study found that a 3-week 17-βE therapy administered in an ω-3-PUFA-encapsulated nanoemulsion setup improved acute vascular damage with just 30% arterial stenosis [109].

Another study conducted by a separate group in 2019 focused on utilizing atorvastatin nano lipid carriers preloaded with ω-3 PUFAs to lower hyperlipidemia. When compared with the commercial formulation, orally administered ω-3-FA-based Atorvastatin reloaded nano lipid carriers resulted in a substantial decrease in low-density lipoprotein and triglyceride levels in the blood [110]. Through various studies, nanoencapsulation has been shown to increase the bioavailability of ω-3 PUFAs in the body. Through their studies, Wakil et al., 2010 and Sanguansri et al., 2013 proved clearly that microencapsulation can improve the availability of FAs [111,112]. 

Nanofibers like zein have also been used to encapsulate ω-3 PUFAs recently, such as in one study by Busolo et al., where 85 wt% DHA-supplemented fish oil was encapsulated in zein through electrospraying assisted by pressurized gas technology (EAPG). The average particle size and encapsulation efficiency were 3.7 ± 1.8 μm and 84%, respectively. The fortified reconstituted milk with zein/DHA-enriched fish oil microcapsules showed no signs of oxidation even after 45 days in an oxidation test [113].

Whey microgels loaded with ω-3 PUFAs were also tested in a study in 2022 where 85 wt% DHA-enriched algal oil was loaded into whey protein microgels by ball milling. The end product had an average particle size of 250 nm, an average diameter of 380 nm, and a polydispersity index of 0.26, indicating the zeta potential. These protein microgels loaded with omega-3 PUFAs addressed several obstacles in the development and storage of omega-3 PUFA oils, such as long-term oxidative resistance and better sensory and textural qualities [114].

A summary of studies utilizing unique nanoparticles for the encapsulation of ω-3 FAs, their mechanism of production, physiochemical properties, and observed effect is listed below in Table 6.

**Figure 3:**
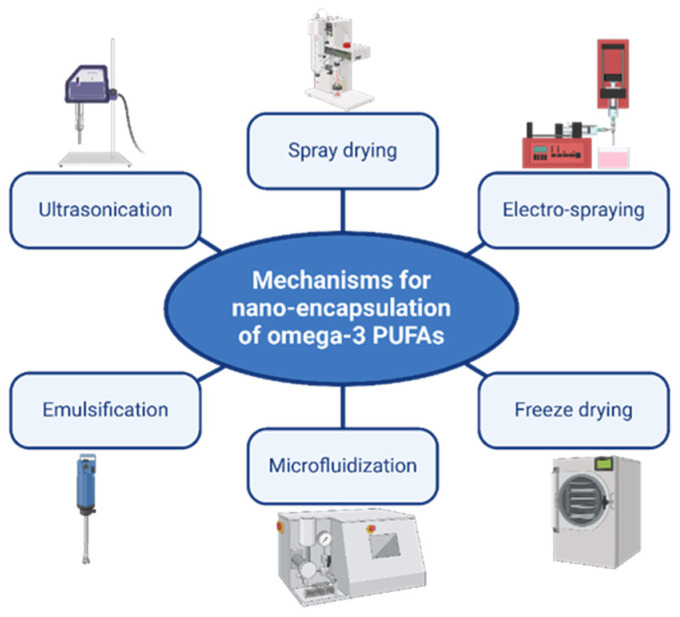
Common physical mechanisms for nanoencapsulation of omega-3 PUFAs.

The most common mechanism for nanoencapsulation of ω-3 FAs in the literature involves physical methods, as illustrated below in Figure 3.

However, lipid-based NPs have their drawbacks as well. Due to their precise crystalline form, they display limited drug loading capacity and the likelihood of drug ejection owing to crystallization during storage [115] along with an initial burst discharge of the drug instead of slow controlled drug release [116]. Other disadvantages during oral administration of lipid-based NPs are the formation of gel of hydrophobic lipid dispersion, restricted loading quantity for hydrophilic formulations, and polymorphic transformation [117]. Liposomes are another type of lipid-based NPs that are employed for drug delivery; however, they tend to have a reduced solubility window [91], problems with drug incorporation and encapsulation, high manufacturing costs, and trouble preserving drug integrity and bioactivity during conjugation [75]. Microgels, on the other hand, are complicated and time-consuming to mass produce on a large scale, as the yield and stability of individual microgels is highly variable using the currently available technology [118]. Nanofibers, although super effective in encapsulating omega-3 PUFAs, have their shortcomings too. Their disadvantages include quick disintegration, low mechanical durability, and full dissolution. Therefore, such fibers must be cross-linked to limit their solubility [119]. 

**Figure 4:**
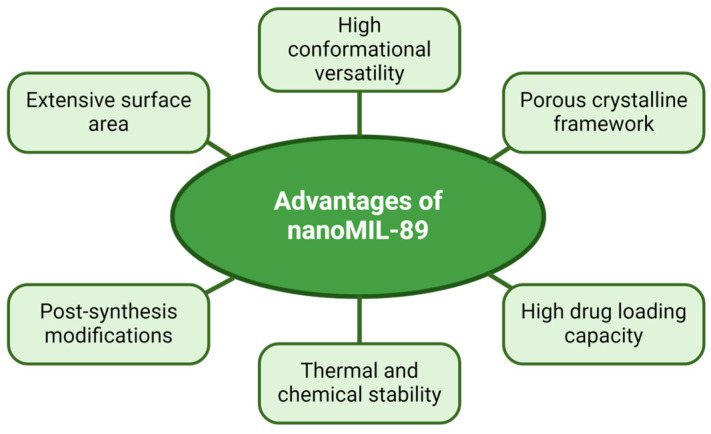
Advantageous features of a type of MOF; Material Institute Lavoisier 89 nanoparticles (nanoMIL-89). Note: these advantages were based on the study by Al-Ansari et al. [124] that addressed the internalization of MOF NPs in human vascular cells.

An alternative to avoid this uncontrolled release would be to use metal–organic framework (MOF) NPs, a novel group of composite nanomaterials, consisting of a combination of inorganic and crystalline organic components [120]. A few of the properties of MOF NPs are their extensive surface area (hollowed-out interior structure) [121], a high degree of porosity, configurable pore dimensions, heat resistance, chemical stability [122], and post-synthesis alterations; these features elevate MOF NPs over lipid-based NPs when it comes to versatility, adaptability, and customizability [123,124]. MOF NPs, such as Material Institute Lavoisier 89 nanoparticles (nanoMIL-89), due to their vast list of perks (Figure 4), can mitigate non-specific drug administration to unsuitable sites, pre-activation of therapeutic agents before reaching the targeted tissue, early immune system approval, and, in certain instances, potentially enhance the pharmacokinetics of drugs at the level of permeability, intake, and dispersion of a drug in the tissue layers [125] (Table 7).

Keeping the hydrophobic nature of omega-3 FAs in mind, the most crucial design consideration in MOF NPs is to modify the interior of the NPs from hydrophilic to hydrophobic. There are two main approaches to solving the issue of interior hydrophilicity. The first is grafting hydrophobic polymers into the interior wall of the NP, which involves linking hydrophobic polymer chains to the interior of MOF pores to alter the interior for improved encapsulation of omega-3 FAs [131]. The second approach incorporates hydrophobic functional groups within the interior, wherein the MOF linkers are chemically modified to contain hydrophobic groups such as isobutyl, alkyl, and isopropyl functional groups [132]. 

The possible administration routes of MOF NPs loaded with omega-3 FAs are specific to the system of choice, which, in this review paper, is the cardiovascular system. There are three possible administration routes to focus on for drug delivery to the cardiovascular system: oral, intravenous, and inhalation routes. However, since inhalation relates more to pulmonary ailments [133] and not directly to cardiovascular disease, the former two routes are preferable. Muchow et al., in their study, developed lipid-based omega-3 FA NPs that proved to be patient-friendly and were administered orally [134]. Another consideration for the oral administration of NPs is coating the NP with polymer substances to protect it from the harsh and acidic gastric environment, for instance, coating NPs with poly lactic-co-glycolic acid (PLGA) [135] and chitosan [136]. Omega-3 FA NPs have also successfully been delivered intravenously in some studies [110], resulting in increased hyperlipidemic action. 

Based on the toxicology of metal-organic framework MIL-89 nanoparticles on embryonic zebrafish development, Al-Ansari et al. said, “The investigation demonstrates that nanoMIL-89 has no developmental harm on zebrafish embryos at low doses (1–10 μM). High concentrations of nanoMIL-89 (>30 μM) significantly impacted hatching time and heart development. The study proves the safety of nanoMIL89 in biological, environmental, and medicinal applications without cytotoxicity beyond a certain concentration of 30μM” [124]. 

Other NPs similar to nanoMIL-89, such as MIL88A and MIL101, are also used for the biomedical application of drug delivery. Since both MIL88A and MIL101 share similar features such as small size, high biocompatibility, etc. [137], to nanoMIL-89, they have also been extensively studied for carrying anti-cancer drugs such as curcumin [138] and doxorubicin [139]. 

**Figure 5:**
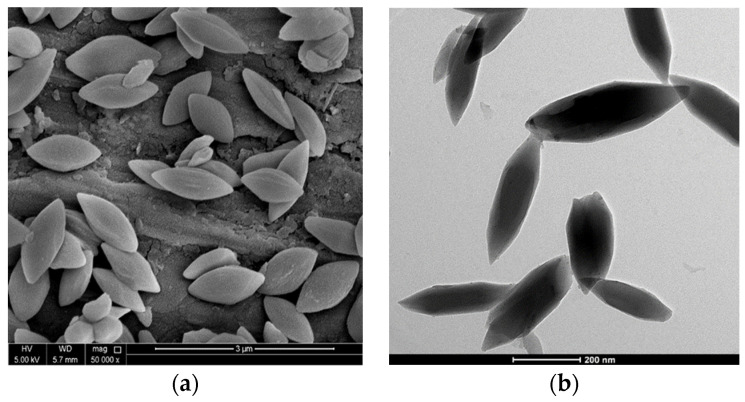
NanoMIL-89 (iron oxide nanoparticles), a subcategory of MOF nanoparticles. (**a**) A scanning electron microscope captured an image of nanoMIL-89 (50,000×). (**b**) A transmission electron microscope captured an image of nanoMIL-89 (5000×).

SEM and TEM analysis is commonly used to characterize the shape and size of nanoparticles after production, as in the case of nanoMIL-89 below (Figure 5).

Paclitaxel (PTX) in microneedle arrays (PTX-MNAs) has higher anticancer efficacy than free PTX (Taxol) in both in vitro and in vivo experiments [140]. Chen and Feng’s research shows that uncoated gold nanoparticles (GNPs) have the potential for skin applications such as penetration, medication loading/release, and combination with physical procedures to treat skin ailments [141]. Gao et al. show that copper sulfide nanoparticles (CuS-TRPV1) can operate as a photothermal switch to minimize atherosclerosis, assisting in cardiac imaging and lowering plaque development in mice, with no long-term damage [142]. Spivak et al. utilized gold NPs loaded with levosimendan (Simdax^®^) in an in vivo study with Wistar rats and concluded that conjugated AuNPs-Simdax^®^ had a favorable impact on cardiac contractile capacity. Additionally, IV administration of 30 nm AuNPs resulted in accumulation in the endothelial cells of infarcted arteries and capillaries. Necrobiosis and fibrosis were greatly reduced following all treatments. Conjugate (Simdax + AuNPs) injections resulted in a considerably larger hydrothorax reduction compared to Simdax injections alone (*p* < 0.01), along with improved cardiac contractile ability. Interestingly, AuNP administration yielded results similar to that of the conjugate [143]. In 2020, Li et al. successfully demonstrated that a gold-nanorod-based NP that catalyzes continuous NO production safeguards against cardiovascular damage in vitro [144]. Some studies employ a hybrid NP that combines liposomes coated with metal NPs, as in the case of Bejarano et al., who developed a gold NP-based nanosystem and employed it to optimize the distribution of angiotensin-1–9, which is a cardioprotectant peptide, to the myocardium, helping both hypertension and myocardial remodeling [145]. Hussein et al. were successful in producing ZnO and gum NPs loaded with 400 mg of DHA solution using a one-step solid-state process [146]. A total of 250 mg of DHA were loaded into ZnO NPs using ultrasonication and homogenization methods [147]. Later, the same researcher also synthesized DHA-loaded AgNPs through nanoprecipitation for antidiabetic drug testing [148]. In a study by El-Daly et al., both DHA-Ag NPs and DHA-ZnO NPs were tested side by side to study the expression of the glucose transport cascade [149,150]. Lastly, another study implied that an administration of 400 µg/kg/day gold NPs helps improve myocardial damage induced by isoproterenol in male albino rats [151]. 

Even though MOF NPs have an array of advantages in their applications, they also come with some drawbacks, such as some of the techniques used to synthesize NPs. The solvothermal and microemulsion techniques are highly expensive. The microemulsion technique uses certain surfactants that are classified as pollutants in the environment. The mechanochemical technique requires a large amount of energy and yields amorphous, not crystalline, NPs, which further cannot be used in X-ray crystal structure analysis. Synthetic techniques for the synthesis of MOFs with effective carbon dioxide absorption capability, such as amine scrubbing, include drawbacks such as high energy usage [152]. Rare-earth-based MOF NPs, namely uranium-, cerium-, lanthanum-, and yttrium-based NPs, are highly expensive, difficult to access, and radioactive, hence dangerous [153]. To conclude, while MOF NPs provide appealing advantages in a variety of applications, their possible downsides highlight the need for additional research and careful evaluation of their usage to maximize their benefits while mitigating related impediments.

## 3. Conclusions

In conclusion, encapsulating omega-3 fatty acids with nanoparticles offers a potential strategy for improving the vital nutrients’ durability, bioavailability, and targeted administration. Investigators have effectively mitigated oxidation, undesirable taste, and restricted GI absorption linked to free omega-3 fatty acids using various encapsulating strategies, from nanoemulsions to solid lipid and polymeric nanoparticles. Nanoparticle containment has various benefits, including environmental protection, precise release dynamics, and the opportunity to integrate other bioactive substances for synergistic effects. Without a doubt, nanoparticles also have drawbacks that need to be addressed to improve acceptability. Furthermore, the nanoscale dimension of these delivery methods facilitates effective cellular absorption and transit beyond biological barriers, resulting in better therapeutic effects.

Nonetheless, additional investigation is needed to optimize the formulation characteristics of nanoparticles, such as structure, dimension, surface characteristics, and encapsulation efficacy, to maximize the bioavailability and effectiveness of omega-3s. Furthermore, long-term stability trials and in vivo tests are required to evaluate the safety, pharmaceutical kinetics, and medicinal potential of nanoparticle-based omega-3 nanoparticles in a variety of clinical contexts. In summary, applying nanoparticles to encapsulate omega-3 fatty acids shows considerable potential for resolving present problems in omega-3 supplementation, while opening up new options for personalized nutrition and preventative healthcare.

## Figures and Tables

**Table 1:** Summary of outcomes of pre-clinical animal studies published between 1992 and 2022 on cardiovascular health utilizing diets rich in omega-3 fatty acids (ω-3 FAs). (↓ indicates a positive decrease in outcome). REF = reference or control diet, SF = sheep fat diet, PUFA = polyunsaturated fatty acids, SSO = sunflower seed oil diet, TFO = tuna fish oil diet, EPA = eicosapentaenoic acid, DHA = docosahexaenoic acid, LNA = Linoleic acid.

Animal Model	Sample Size (*n*)	Omega-3 FA Diet/Dose	Treatment Period	Outcomes	References
Marmoset monkey	29	REF diet: low fat (11.2 % of total energy containing 37.3% saturated fatty acids and 18.3% PUFA) SF diet: sheep fat (48.5 % saturated fatty acids and 12.3% PUFA) SSO diet: sunflower seed oil (23.3% saturated fatty acids and 53.5% PUFA) TFO diet: tuna fish oil (9.3% saturated fatty acids and 33.6% PUFA, with 22.8% n-3 PUFA)	30 months	↓ in the threshold for ventricular fibrillation during acute myocardial ischemia induced by coronary artery occlusion but it remained higher in the PUFA-fed animals.	[24]
Mongrel dogs (dog model of sudden cardiac death; anterior wall myocardial infarction produced surgically, and an inflatable cuff placed around the left circumflex coronary artery)	17	EPA intravenous administration (5 mL) (*n* = 7, 20:5 ω-3; 98.4% free EPA, 1.1% free DHA) DHA intravenous administration (5 mL) (*n* = 8, 22:6 ω-3; 90.8% free DHA, 0.9% EPA) LNA intravenous administration (5 mL) (*n* = 8, >99% LNA) 4 of 7 EPA tests and 5 of 8 DHA trials used the same dose (0.86 g carried in albumin). In the other three tests using EPA or DHA, 5 mL of the pure fatty acid was delivered as egg lecithin emulsions.	1 week	↓ in ventricular flutter-fibrillation. EPA, DHA, and LNA substantially decreased the incidence of ventricular flutter-fibrillation, protecting 5 of 7 (*p* = 0.0105), 6 of 8 (*p* = 0.0035), and 6 of 8 animals, respectively, whereas the control lipid emulsion of triglycerides of soybean oil containing around 7% to 8% esterified LNA (Intralipid, *n* = 7) failed to safeguard any animal from malignant arrhythmias.	[25]
Rats	115 (Male, 3-month-old)	No omega-3 PUFA EPA 5 g/kg DHA 5 g/kg EPA + DHA 2.5 g/kg of each EPA + DHA 5 g/kg of each	2 weeks	Substantial reductions (*p* < 0.05) in mean arterial pressure and platelet-rich plasma levels. The EPA and DHA diets reduced infarct size relative to the vehicle (*p* < 0.05), but the EPA + DHA 2.5 g/kg and 5 g/kg diets did not vary substantially from the controls. DHA decreased caspase-3 activity, but EPA and DHA diets significantly increased Akt activity (*p* < 0.05).	[26]
Mice (sickle red cell membrane)	14	Standard rodent diet (soy-diet with n6/n3 ratio of 8:1) ω-3 FD purified rodent diet (2.1% calories from ω-3 oil)	6 weeks	↓ in Endothelin-1 (ET-1) and Vascular Cell Adhesion Molecule-1 (VCAM-1). In sickle cell mice, a fish oil diet resulted in significantly greater mean corpuscular volume and lower mean cell hemoglobin concentration relative to control mice fed with a soy oil diet, but no significant changes in hemoglobin levels.	[27]
Mice	36 (Female mice)	Low (0 g/kg EPA + DHA) or high (12.2 g/kg EPA + DHA) and two chemotherapy models (9 mg/kg anthracycline + 90 mg/kg cyclophosphamide)	2 weeks	↓ in the expression of the myosin heavy chain 7 (Myh7) gene and collagen type III alpha 1 chain (Col3a1). The high LC n-3 PUFA diet also dramatically changed several lipid species in cardiac mitochondrial preparations, including multiple epoxy fatty acids [17(18)-EpETE] and N-acylethanolamines (arachidonoylethanolamine, AEA).	[28]

**Table 2:** Summary of outcomes of randomized clinical trials (RCTs) published between 1989 and 2021 on cardiovascular health utilizing diets rich in ω-3 FAs. (↓ indicates a positive decrease in outcome, **↓** indicates a negative decrease in outcome, and ↑ indicates a negative increase in outcome).

Study Name	Sample Size (*n*)	Omega-3 FA/Fish Dose per Day	Follow-Up Period	Outcomes	References
DART Clinical Trials (1989)	2033 (Men below 70 years with a history of MI)	2–3 servings of fish/week	2 years	**32% ↓** reinfarction, **29% ↓** in 2-year mortality from all causes. Secondary prevention of MI, **3–4% ↓**in serum cholesterol. The 2-year incidence of reinfarction plus death from ischemic heart disease was not significantly affected by any of the dietary regimes. A modest intake of fatty fish (2–3 portions a week) may reduce mortality in men who have recovered from MI.	[29]
GISSI-Prevenzione Trials (1999)	11,324	n-3 PUFA (1 g daily, *n* = 2836) vitamin E (300 mg daily, *n* = 2830) both (*n* = 2830) none (*n* = 2828)	3.5 years	**28% ↓** in mortality from all causes, and **45% ↓** in sudden cardiac death. Treatment with n-3 PUFA, but not vitamin E, significantly lowered the risk of the primary endpoint (relative risk decreased by 10% [95% CI] by two-way analysis, 15% by four-way analysis, and cardiovascular death (17% two-way, 30% four-way)	[30]
JELIS Clinical Trials (2007)	18,645 (Japanese)	1800 mg of EPA daily with statin (EPA group; *n* = 9326) Statin only (10 mg of pravastatin or 5 mg of simvastatin) (controls; *n* = 9319)	5 years	The primary endpoint was detected in 262 (2.8%) patients in the EPA group and 324 (3.5%) in controls—a **19% ↓** in major coronary events (*p* = 0·011). Post-treatment, there was a **25% ↓** in LDL cholesterol concentrations from 4·7 mmol/L in both groups. Unstable angina and non-lethal coronary events were also significantly reduced in the EPA group. In patients with a history of CAD who were given EPA treatment, there was a **19% ↓** in major coronary events. In patients with no history of CAD, who were given EPA treatment, there was an **18% ↓** in major coronary events, but this finding was insignificant.	[31]
DOIT Clinical Trials (2010)	563 (Norwegian men aged 64–74)	Control (no dietary counseling and placebo, *n* = 142), Diet only (dietary counseling and placebo, *n* = 139), n-3 PUFA only (no dietary counseling and n-3 PUFA supplementation, *n* = 140) Combined (dietary counseling and n-3 PUFA supplementation (2.4 g of ω-3 PUFA, *n* = 142)	3 years	**↓** in all-cause mortality and cardiovascular events. The HRs for all-cause mortality and cardiovascular events were 0.57 (95% CI: 0.29–1.10) and 0.86 (0.57–1.38), accordingly.	[32]
OMEGA Trial (2010)	3851 (Patients that had MI 3–14 days prior)	Control (soft gelatin capsule with 1 g olive oil) Treatment (1 g omega-3 acid ethyl esters, 460 mg EPA and 380 mg DHA	1 year	**No ↓** in mortality due to unexpected cardiac events. Sudden cardiac death in survivors of acute myocardial infarction; not only without reduction in mortality, but also during a follow-up of 365 days, the event rates were (omega and control groups) as follows: sudden cardiac death, 1.5% and 1.5% (*p* = 0.84); total mortality, 4.6% and 3.7% (*p* = 0.18); major adverse cerebrovascular and cardiovascular events, 10.4% and 8.8% (*p* = 0.1); and revascularization in survivors, 27.6% and 29.1% (*p* = 0.34).	[33]
Alpha Omega Trial (2010)	4837 (Dutch men and women aged 60–80 years, who were survivors of MI)	Control (Placebo margarine) ALA (2 g) treatment EPA (400 mg) + DHA (400 mg) treatment EPA (400 mg) + DHA (400 mg) +ALA (2 g) treatment	3.3 years	**No ↓** in incidence of serious cardiac events. Throughout the follow-up time frame, a significant cardiovascular incident occurred in 671 individuals (13.9%). Neither EPA–DHA nor ALA decreased this main endpoint (HR with EPA–DHA, 1.01; 95% CI: 0.87 to 1.17; *p* = 0.93; HR with ALA, 0.91; 95% CI: 0.78 to 1.05; *p* = 0.20). In the prespecified subgroup of women, ALA was related with a near-significant decrease in the incidence of major cardiovascular events (HR, 0.73; 95% CI: 0.51 to 1.03; *p* = 0.07).	[34]
SU.FOL.OM3 Trial (2010)	2501 (history of myocardial infarction, unstable angina, or ischemic stroke)	Daily dietary supplement containing 5-methyltetrahydrofolate (560 μg), vitamin B-6 (3 mg), and vitamin B12 (20 μg) or placebo, and containing omega-3 fatty acids (600 mg of eicosapentaenoic acid and docosahexaenoic acid at a ratio of 2:1)	5 years	**No ↓** in incidence of serious cardiovascular events. In the LC-OM3 group, 1613 cardiac deaths were recorded (4.48% of subjects), compared with 1746 cardiac deaths in the control groups (4.87% of subjects).	[35]
ASCEND Trial (2018)	15,480 (individuals with diabetes)	0.41 g eicosapentaenoic acid, 0.34 g docosahexaenoic acid Aspirin and omega-3 (3870) Aspirin and placebo omega-3 (3870) Placebo aspirin and omega-3 (3870) Placebo aspirin and placebo omega-3 (3870)	7.4 years	**No ↓** in major vascular incidents	[36]
VITAL Trial (2018)	25,871 (American men aged 50 years and older and American women aged 55 years and older)	Vitamin D (2000 IU/day) n-3 fatty acids (1 g/day as a fish-oil capsule consisting of 840 mg of n-3 fatty acids, including 460 mg of EPA and 380 mg of DHA)	5.3 years	**No ↓** in risk of cardiovascular diseases. In the evaluations of key secondary endpoints, the risk ratios were: for the expanded composite endpoint of cardiovascular events, 0.93 (95% CI: 0.82 to 1.04); for total MI, 0.72 (95% CI: 0.59 to 0.90); for total stroke, 1.04 (95% CI: 0.83 to 1.31); for death from cardiovascular causes, 0.96 (95% CI: 0.76 to 1.21); and for death from cancer (341 deaths from cancer), 0.97 (95% CI: 0.79 to 1.20).	[37]
REDUCE-IT Trial (2018)	8179 (Patients aged 45 or older with a history of CVD or aged 50 or older with a history of diabetes mellitus)	Placebo (*n* = 4090) 4 g of icosapent ethyl (EPA ethyl ester) (*n* = 4089)	4.9 years	**↓** in the incidence of ischemic events. **18.3% ↓** in triglyceride level from baseline to 1 year in icosapent ethyl group. **3.1% ↑** in LDL cholesterol level from baseline to 1 year in icosapent ethyl group.	[38]
STRENGTH Trial (2020)	13,078	4 g omega-3 CA formulation (EPA + DHA)/day (*n* = 6539) Corn oil (*n* = 6539)	3 years	**No ****↓** in cardiovascular events. The primary endpoint was seen in 785 patients (12.0%) administered with omega-3 CA compared to 795 (12.2%) treated with corn oil (hazard ratio, 0.99 [95% CI: 0.90–1.09]; P = 0.84). The omega-3 CA group reported more gastrointestinal side events (24.7%) than maize-oil-treated individuals (14.7%).	[39]
OMEMI Trial (2021)	1027 (patients aged 70 to 82 years with recent (2–8 weeks) AMI)	Placebo (corn oil; 56% linoleic acid, 32% oleic acid, 10% palmitic acid). 1.8 g ω-3 FAs (930 mg EPA + 660 mg DHA)	2 years	**No ↓** in the frequency of cardiovascular events or deaths from all causes. The primary endpoint was a composite of nonfatal AMI, unscheduled revascularization, stroke, all-cause death, and heart failure hospitalization after 2 years. The secondary outcome was new atrial fibrillation. The safety outcome was major bleeding.	[40]

**Table 3:** Summary of outcomes of cohort studies published between 2000 and 2021 on cardiovascular health utilizing diets rich in ω-3 FAs. (↓ indicates a positive decrease in outcome, **↓** indicates a negative decrease in outcome, and ↑ indicates a negative increase in outcome).

Study Name and Year	Sample Size (*n*)	Diet (Consisting of ω-3 FA)	Follow-Up Period	Outcomes	References
NHANES I Trial (1971–1994)	8825 (7421 people and 1404 black people aged 25–74 years with no CVD history)	1 serving of fish/week >1 serving of fish/week	22.1 years	**No ↓** in risk of cardiovascular disease (CVD) after consumption of 1 serving of fish/week (relative risk 0.76, 95% CI: 0.63–0.91). There was no further risk decrease observed for >1 serving of fish consumed weekly (relative risk 0.85, 95% CI: 0.68–1.06). Other main outcomes measured were death (all causes, cardiovascular, non-cardiovascular, cancer) and incidence of CHD.	[42]
JPHC Study (1992–2001)	41,578 (19,985 and 21,593 Japanese men and women aged 40–59 years with no history of CVD)	180 g serving fish/day	9 years	**40% ↓** in the incidence of CHD, specifically MI and nonfatal CHD among people in the highest percentile of fish consumption (180 g/day) as compared to a moderate fish diet (23 g/day).	[43]
EPIC-Germany (1994–1998)	48,315 (Caucasian people aged 35–65 years)	<7.5 g fish/day 7.5-14.5 g fish/day 14.5-21.5 g fish/day 21.5-31.1 g fish/day >31.3 g fish/day	8.1 years	**No ↓** in risk of myocardial infarction (MI) or stroke. In all the quartiles, fish intake did not increase the incidence of MI (HR 0.84, 95% CI: 0.66, 1.08, *p* = 0.21), or fatal MI (HR 1.18, 95% CI: 0.68, 2.06, *p* = 0.37). There was no significant negative correlation with non-fatal MI (HR 0.78, 95% CI: 0.59-1.03, *p* = 0.07). Fish intake did not increase the risk of total stroke (HR 0.96, 95% CI: 0.73, 1.26, *p* value= 0.67), ischemic stroke (HR 0.87, 95% CI: 0.64, 1.19, *p* value= 0·66), or hemorrhagic stroke (HR 1.46, 95% CI: 0.77, 2.78, *p* value = 0.16).	[44]
REGARDS Study (2004–2007)	16,479 (58% white, 42% African American, 55% female, and 45% male.)	>2 servings of fried fish/week	5.1 years	↑ in the chance of cardiovascular events. Participants who had ≥2 servings of fried fish per week had a substantially higher risk of cardiovascular events (HR = 1.63; 95% CI: 1.11–2.40). The consumption of non-fried fish was not related to an increased risk of incident CVD. There was no link established between dietary fried or non-fried fish consumption and cardiovascular or all-cause death.	[45]
Moli-Sani Study (2005–2010)	20,969 (without a history of CVDs)	0–2 servings of fish/week 2–4 servings of fish/week >4 servings of fish/week	4.3 years	**40% ↓** in the likelihood of CHD (HR = 0.60; 95% CI: 0.38–0.94) and stroke (HR = 0.60; 95% CI: 0.40–0.90) in those consuming >4 servings of fish weekly.	[46]
EPIC-NL (1993–2011)	34,033 (Dutch participants)	0 serving of fish/week <1 serving of fish/week ≥1 serving of fish/week	18 years	**↓** in risk of ischemic stroke (HR: 0.70, 95% CI: 0.57–0.86) in those who consumed ≥1 serving of fish/week compared to non-fish consumers.	[47]
MVP Study (2011–2017)	197,761 (92% male and 8% female participants with no history of stroke or CAD)	<1 serving of fish/month 1–3 servings of fish/month 1 serving of fish/week 2–4 servings of fish/week 5–6 servings of fish/week 1 serving of fish/day 2–3 servings of fish/day 4–5 servings of fish/day >6 servings of fish/day	3.3 years	**↓** in risk of non-lethal ischemic stroke. Omega-3 fatty acid supplementation was independently linked to a decreased incidence of non-lethal ischemic stroke [HR (95% CI): 0.88 (0.81, 0.95)] but not non-lethal CAD [0.99 (0.93, 1.06)]. Consumption of fish was not linked with non-lethal CAD [1.01 (0.94, 1.09) for 1–3 servings/month, 1.03 (0.98, 1.11) for 1 serving/week, 1.02 (0.93, 1.11) for 2–4 servings/week, and 1.15 (0.98, 1.35) for ≥5 servings/week] or non-lethal ischemic stroke [0.92 (0.84, 1.00) for 1–3 servings/month, 0.93 (0.85, 1.02) for 1 serving/week, 0.96 (0.86, 1.07) for 2–4 servings/week, and 1.13 (0.93–1.38) for ≥5 servings/week].	[48]
Alpha Omega Study follow-up (2006–2018)	4067 (Dutch patients aged 60–80 years with a history of MI)	≤50 mg ω-3 FA/day >50–100 mg ω-3 FA/day >100–200 mg ω-3 FA/day 200 mg ω-3 FA/day	12 years	**30% ↓** in the possibility of deadly CHD. The dietary intake of EPA + DHA was substantially inversely linked with only CHD mortality (HR, 0.69 [0.52–0.90] for >200 mg/day vs. ≤50 mg/d; HR, 0.92 [0.86–0.98]). Similar findings were reported for fish intake (HRCHD, 0.74 [0.53–1.03] for >40 mg/day versus ≤5 g/d; *p* value = 0.031). Circulating EPA + DHA was inversely linked with CHD mortality (HR, 0.71 [0.53–0.94]) as well as CVDs and all-cause mortality.	[49]

**Table 4:** Summary of outcomes of case-control studies published between 1990 and 2010 on cardiovascular health utilizing diets rich in ω-3 FAs. (↓ indicates a positive decrease in outcome.)

Year of Study	Sample Size (*n*)	ω-3 FA in Fish Consumption	Study Period	Outcomes	References
1985–1990	287 (women with acute MI)	1 serving of fish/week	5 years	**↓** the likelihood of cardiovascular diseases (CVDs). Significant inverse relationships were identified for fish with an odds ratio of **0.6** for acute MI.	[53]
1988–1994	334 (aged between 25 and 74 years)	5.5 g/month	6 years	**50% ↓** in the possibility of first cardiac arrest. An RBC membrane n-3 polyunsaturated fatty acid percentage of 3.3% of total fatty acids was related to a **70%** reduction in the likelihood of primary cardiac arrest (OR, 0.3; 95% CI: 0.2 to 0.6).	[54]
1994	78 (aged 30–60 years)	>1 fish serving/week <1 fish serving/week	18 months	**↓** in risk of myocardial infarction (MI) in those consuming >1 fish serving/week. Subjects who had at least one supper of fish per week had substantially greater levels of Ery-Hg and P-PUFA, but not Ery-GSH-Px (*p* < 0.001). This implies that Ery-Hg and P-PUFA reflect prior long-term fish consumption. High Ery-Hg and P-PUFA levels were related to a lower risk of MI. In a multivariate analysis, those with high Ery-Hg and P-PUFA had a lower risk of myocardial infarction (OD 0:16, 95% CI: 0:04, 0:65).	[55]
2000–2001	848 (695 males and 153 females with first diagnosed ACS event)	<150 g fish/week 150–300 g/week >300 g/week	1 year	**38% ↓** in the likelihood of developing acute coronary syndrome (ACS). <150 g fish consumption/week was linked to a **12%** (OD 0.88, *p*-value < 0.05) reduced risk of having ACS in men and **26%** (OD = 0.74, *p*-value < 0.05) reduced risk in women. However, intermediate (150–300 g/week) and high (>300 g/week) fish intake failed to affect disease development (OD = 1.10 and 1.01, respectively, *p*-value > 0.1).	[56]
2004–2006	108	2 servings of fish/week	2 years	**↓** in risk of coronary events. The odds ratio (CI 95%) for developing CAD after eating fish was lower [**0.55** (0.31–0.91)] than that of other consumables.	[57]

**Table 5:** Characteristics, uses, advantages, and disadvantages of different classes of nanoparticles.

Nanoparticle Type	Characteristics	Advantages	Disadvantages	Application/Use	References
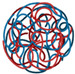 Polymeric NPs (organic)	Solid particles (colloidal) that range in size from 10 to 100 nm. Two main types; nanocapsules and nanospheres.	Controlled and sustained drug release, stable, and efficient. Biodegradable, good biocompatibility.	Difficult to scale up, lack of toxicological evaluation, can be an environmental hazard and can pose an occupational hazard during production.	Vaccine delivery, cancer treatment, antibiotic delivery, purification of biomolecules, and bioimaging.	[79,80,81]
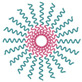 Polymeric micelles (organic)	Spherical, 10–100 nm in diameter. Amphiphilic block copolymers produce nanoscopic core/shell structures via covalent bonding. Hydrophobic core, hydrophilic shell.	Highly stable, high loading efficiency, selective and controlled drug release, and kinetically stable.	Low solubility, low loading capacity, low stability in vivo, and can dissociate in vivo.	Cancer treatment, food-based technology, drug delivery, photodynamic therapy, and gene delivery.	[82,83,84]
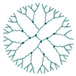 Dendrimer NPs (organic)	Spherical, compact, 1–100 nm in diameter. Comprises a central core atom, followed by repeating branching subunits and terminal groups.	High loading capacity, high bioavailability, high penetrability, high symmetry, and surface groups can be customized easily.	Low water solubility, high nonspecific toxicity, challenging to separate the NPs from the reactants, and time consuming.	Biomedical applications, targeted delivery, cancer treatment, cancer diagnosis, and antibacterial therapy.	[85,86,87]
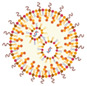 Solid lipid NPs (organic)	Spherical shape, diameter ranging from 50 nm to 1 μm, big surface area, substantial drug loading capacity, and surfactant on the outer layer.	Controlled and/or targeted medication release, optimized drug stability, higher and improved drug content, and non-toxic.	Drug ejection upon polymeric transformation during storage and high moisture content of the dispersions.	Gene vector transporter, topical drug application, cosmetics, agricultural usage, and an anticancer medication carrier.	[88,89]
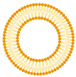 Liposomes (organic)	Spherical lipid vesicles 50–500 nm in diameter comprised of several lipid bilayers formed by the emulsification of real or artificial lipids in water-based solutions.	Improved effectiveness, improved therapeutic value of drugs, improved stability by encapsulation, not toxic, adaptive, and biocompatible.	Poor solubility, transient half-life, oxidation, hydrolysis, leak, coagulation of enclosed molecules, and expensive manufacturing.	Anticancer drug delivery, antifungal drug delivery, analgesic delivery, COVID-19 mRNA vaccines, and photodynamic therapy.	[90,91]
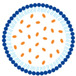 Nanoemulsion (organic)	Spherical, 20–500 nm diameter, 10–20% polydispersity, unstable thermodynamically, stable kinetically.	Large surface area, high free energy, manufactured in an array of formulations, not toxic, and a nonirritant.	Stabilization requires a high concentration of surfactant, and stability is regulated by pH and temperature.	Cosmetics, food, pharmaceuticals, drug delivery, vaccine delivery, material synthesis, and encapsulation of natural food preservatives.	[92,93]
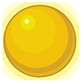 Gold NP (inorganic)	Spherical, 10–100 nm in diameter, colored orange, brown, red, or purple, and absorbs between 500 and 550 nm.	High surface area to volume proportion, very stable, good biocompatibility, customizable, steady size and shape.	Gold NPs can be toxic in large doses. Gold NPs entrapped in the liver might impair its function and manufacturing is costly.	Imaging, electronic gadgets, material production, colorimetric and electrochemical sensing, drug delivery, and cancer diagnosis.	[94,95]
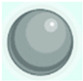 Silver NP (organic)	Various shapes (spherical, triangular, hexagonal, octagonal, etc.), 1–100 nm diameter, small size crystalline, high heat conductivity, and high electric conductivity.	High surface area, bactericidal, catalytic features, fungicidal, not toxic, anticancer properties, very stable, and high solubility.	Limited resolution, numerous light scatterings, sedimentation, and high energy required in preparation.	Disease diagnosis, agriculture, cosmetics biotechnology, wound dressing, textile industry, and antiseptic reagents.	[96,97]
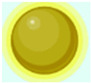 Iron oxide NP (inorganic)	Various shapes (spherical, cubes, hexagonal, rods, etc.), superparamagnetic, 10–20 nm in diameter or less, different forms such as hematite, magnetite, and maghemite.	High surface area to volume proportion, inexpensive, low toxicity, high binding capability, substantial dispersibility, not toxic.	Highly reactive, agglomerate, surface oxidation, absence of functional groups, reduced capacity to adsorb molecules, slow kinetics, leach in low pH.	Biomedical, magnetic resonance imaging diagnosis, drug delivery, antibody and vaccine manufacture, gene therapy, cancer therapy, and sensory probes.	[98,99,100,101]
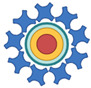 Quantum dots (inorganic)	Various shapes (spherical, cuboidal, conical, etc.), 2–20 nm in diameter, metallic or semi-conductors, can be zero, one, two, or three dimensional, nanocrystals, and have 100–10000 atoms and <100 electrons.	Customizable morphology, great biocompatibility, high ability to disperse, magnetic, and great optical features.	Toxic, lacks significant polarization, water insolubility, and needs strong polymer casing.	Photocatalysis, biosensing, bioimaging in vivo and in vitro, optoelectrical gadgets, and microscopy.	[102,103]
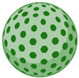 Mesoporous NP (inorganic)	Spherical or rod-shaped, 30–300 nm in diameter, majorly made up of silicone, highly structured pores, stable porous matrix, five different types of nanocomposites.	Low toxicity, high biocompatibility, large surface area, big pore volume, heat stable, chemically stable, customizable pore size.	Mild toxicity, silanol moieties on the surface can interact with the outermost layer of red blood cell membrane phospholipids causing hemolysis and induction of metabolic alterations promoting melanoma.	Cancer treatment, biosensing, bioimaging, targeted illness treatment, radiotherapy, chemotherapy, dynamic therapy, thermal therapy, immune therapy, and gene therapy.	[104,105,106]

**Table 6:** Summary of studies encapsulating omega-3 fatty acids using different types of nanoparticles between 2013 and 2022 prepared by a plethora of different techniques and having varying physiochemical properties.

Omega-3 Dose and Source	Nanoparticle Type	Production Technique	Physiochemical Characteristics	Effect	References
Flaxseed oil 20%, *w*/*v*	Nanoemulsion	Microfluidization	Average diameter = 146 nm Surface charge = 34 mV Encapsulation efficiency = 93% and 99%	Strong anti-proliferative impact on vascular smooth muscle cells	[108]
Flaxseed oil 20%, *w*/*v*	Nanoemulsion	Microfluidization	Average diameter = 187 ± 7.5 nm and 176 ± 4.8 nm Surface charge = (−54.6 ± 4.1 mV and -56.4 ± 5.1 mV) Encapsulation efficiency = 94.6%	Improved acute vascular damage with only 30% arterial stenosis	[109]
Omega-3 FA	Atorvastatin-loaded nano lipid carrier	Melt emulsification and ultrasonication method	Particle size = 87.29 ± 6.68 nm Surface charge = −36.03 ± 1.50 mV Encapsulation efficiency = 86.70% ± 0.15	Improving omega-3 FA bioavailability and antihyperlipidemic action	[110]
85 wt% Docosahexaenoic-acid-supplemented fish oil	Nanofiber	Electrospraying assisted by pressurized gas technology (EAPG)	Average particle size = 3.7 ± 1.8 μm Encapsulation efficiency = 84%	Supplemented reconstituted milk with zein/DHA-enriched fish oil microcapsules showed no signs of oxidation even after 45 days.	[113]
85 wt% DHA enriched algal oil	Oleogel-based microgel	Ball milling	Whey protein microgel particle size = 250 nm Polydispersity index = 0.29 Diameter = 380 nm	Protein microgels addressed various obstacles in the development of omega-3 polyunsaturated fatty acid oils, such as long-term oxidative resistance and better sensory and textural qualities.	[114]

**Table 7:** Physiochemical properties and related biomedical advantages of MOF NPs.

Physiochemical Properties of MOF NPs	Related Biomedical Advantages	References
High surface area compared to volume	Ability to make post-synthesis surface modifications	[121,126]
Biocompatibility	Reduced physiological toxicity	[127]
Small size	Higher bioavailability and higher penetrability	[125,128]
A high degree of porosity	Higher drug loading capability	[122,129]
Presence of various functional groups	Increased mechanical stability and increased thermal stability	[123,130]
